# Factor analysis for construct validity of a trunk impairment scale in Parkinson’s disease: a cross-sectional study

**DOI:** 10.3389/fneur.2023.1303215

**Published:** 2024-01-03

**Authors:** Kazunori Sato, Yuta Yamazaki, Yoshihiro Kameyama, Koji Watanabe, Eriko Kitahara, Koshiro Haruyama, Yoko Takahashi, Yuji Fujino, Tomofumi Yamaguchi, Tadamitsu Matsuda, Hitoshi Makabe, Reina Isayama, Yuhei Murakami, Mami Tani, Kaoru Honaga, Kozo Hatori, Yutaka Oji, Yuji Tomizawa, Taku Hatano, Nobutaka Hattori, Toshiyuki Fujiwara

**Affiliations:** ^1^Department of Rehabilitation Medicine, Juntendo University Graduate School of Medicine, Tokyo, Japan; ^2^Department of Physical Therapy, Faculty of Health Science, Juntendo University, Tokyo, Japan; ^3^Department of Neurology, Juntendo University Hospital, Tokyo, Japan

**Keywords:** Parkinson’s disease, trunk impairment, rehabilitation, physical therapy, factor analysis

## Abstract

**Objectives:**

To investigate the construct validity of the Trunk Impairment Scale (TIS), which was developed to assess trunk impairment in patients with stroke, in patients with Parkinson’s disease (PD).

**Design:**

This retrospective, cross-sectional study enrolled consecutive PD inpatients. Correlation analysis was performed to clarify whether the TIS assessment was related to other balance functions, lower extremity muscle strength, or walking ability. Factor analysis was performed to see how the background factors of TIS differ from balance function, lower limb muscle strength, and walking ability.

**Results:**

Examining the data of 471 patients with PD, there were relationships between TIS and the Mini-Balance Evaluation Systems Test (*r* = 0.67), Barthel Index (*r* = 0.57), general lower limb extension torque (*r* = 0.51), two-minute walk test (*r* = 0.54), Hoehn and Yahr stage (*r* = −0.61), and Movement Disorder Society Unified Parkinson’s Disease Rating Scale part III total points (*r* = −0.59). Factor analysis showed that TIS items were divided into three factors (an abdominal muscles and righting reflex component; a perception and verticality component; and a rotational component), differing from other scales that included clinical assessment items.

**Conclusion:**

The TIS can be useful for assessing the underlying trunk impairment as a basis for activities of daily living, gait function, and balance ability in patients with PD.

## Introduction

Parkinson’s disease (PD) is a progressive neurodegenerative disease with loss of dopaminergic neurons in the substantia nigra pars compacta that causes bradykinesia, muscle rigidity, and resting tremor. Patients with PD also have axial symptoms, such as postural instability, gait disturbance, and postural abnormalities, during the advanced stages of the disease (1). Factors thought to cause axial symptoms include lower extremity muscle weakness, bradykinesia, rigidity, abnormal pattern of postural reflexes, dystonia, reduced gait automaticity, impaired proprioception, and impaired cognitive function, as well as poor trunk function (2–5). It is also known that trunk impairment is often observed (6).

Previous studies reported that trunk impairment is associated with postural instability, postural abnormality, and gait disturbance that reduce quality of life in patients with PD (3, 7–9). However, a battery of tests for trunk impairment focused on the trunk angle in one plane or the motor function and mobility of the trunk, and these studies separated each other. Moreover, they did not include an assessment of the perceptual aspect (6, 10–13). Proprioception and sensory integration are crucial for trunk function, but they were found to be impaired in patients with PD (14–16). Therefore, a comprehensive evaluation of trunk impairment for patients with PD should be a single assessment battery and include the perceptual aspect, in addition to the motor function and trunk deviation angle.

The Trunk Impairment Scale (TIS) reported by Fujiwara et al. (17) was originally developed to comprehensively assess the trunk impairment of patients with stroke based on the motor and perceptual aspects with seven items (1. perception of trunk verticality; 2, 3. trunk rotation muscle strength on the right and left sides; 4, 5. righting reflex on the right and left sides; 6. verticality; and 7. abdominal muscle strength). The TIS had good reliability and validity in patients with stroke (17). The TIS can predict functional prognosis in patients with early stroke and is an indicator of functional improvement in patients with PD (18, 19). However, the construct validity of TIS reported by Fujiwara has not been examined in patients with PD. This study aimed to assess the trunk impairment of patients with PD using the TIS reported by Fujiwara and to confirm its construct validity. Another objective was to analyze how the TIS is related to disease severity and various physical functions and to determine its clinical usefulness.

## Methods

### Study design

This retrospective, cross-sectional study was conducted with the approval of the Research Ethics Committee, Faculty of Medicine, Juntendo University (E22-0157-H01). Informed consent was obtained in the form of an opt-out on the website. This study is reported following the STrengthening the Reporting of OBservational studies in Epidemiology (STROBE) statement (20).

No previous study reported trunk function in PD including perceptual aspects in its assessment items without TIS reported by Fujiwara; therefore, a sample size calculation for analyzing correlations was considered based on a small effect size (*r* = 0.2), significance level of 0.01, and power of 0.95 to minimize the probability of committing type II error (21). Sample size calculations were performed with G*Power 3.1 (21). Since the medical record data from regular practice are expected to contain many deficiencies, over 431 patients’ medical record data were included in the study.

### Study subjects

A total of 482 consecutive patients with PD of all ages who were admitted to the hospital from January 2019 to April 2022 and underwent rehabilitation were included in the study. The patients had been clinically diagnosed with established and probable Parkinson’s disease according to the Movement Disorder Society Clinical Diagnostic Criteria (1) by neurologists specialized in movement disorders.

The exclusion criteria included patients who were diagnosed with other neurodegenerative diseases (e.g., progressive supranuclear palsy or multiple system atrophy), had complications that severely impaired their physical performance (e.g., orthopedic disease, internal disease, or psychiatric disease), or patients who refused to participate in this research project. In addition, patients with significant missing records were also excluded.

### Assessment items

Based on updated medical records when patients with PD visited the hospital, the data of age, sex, duration of disease, duration of levodopa medication, body mass index, Hoehn and Yahr stage, Levodopa Daily Dose (LDD), and Levodopa Equivalent Daily Dose (LEDD) were collected. Part II-Activity Daily living items of the Movement Disorder Society Unified Parkinson’s Disease Rating Scale (MDS-UPDRS) and Highest ON medication state of part III-Motor items of MDS-UPDRS were collected from updated medical records by neurologists specialized in movement disorders. The physical functions of the TIS, Barthel Index, Mini-Balance Evaluation Systems Test (Mini-BESTest), two-minute walk test, general lower extremity extension torque using the Strength Ergo 240, and handgrip force were assessed by physical therapists. The assessments of physical function were performed 60 to 120 min after oral dosing with confirmed ON-medication status. Each assessment for the physical functions is described in detail below.

### Trunk impairment scale

The TIS consists of seven items: (1) perception of trunk verticality; (2, 3) trunk rotation muscle strength on the right and left sides; (4, 5) righting reflex on the right and left sides; (6) verticality; and (7) abdominal muscle strength. Each item is evaluated on a scale of 0 to 3, with a maximum score of 21 points; a higher score represents better trunk function. The required duration for the completion of the TIS is approximately 5 min. The Appendix shows the details of the TIS’s definition, procedures, and scoring criteria (17).

### Postural instability and gait dysfunction

Postural instability and gait dysfunction (PIGD) was assessed with the PIGD sub-score of MDS-UPDRS part III (item 3.9 Arising from chair, 3.10 Gait, 3.11 Freezing of gait, 3.12 Postural stability, 3.13 Posture) (22).

Specific balance abilities were measured with the Mini-Balance Evaluation Systems Test (Mini-BESTest), which consists of four different balance aspects (Anticipatory postural adjustments, Automatic postural responses, Sensory orientation, and Dynamic gait) with the highest score of 28 points (23); higher scores indicate better balance ability. Gait performance was assessed with a two-minute walk test (24, 25).

### Strength of the upper and lower extremities and foot bradykinesia

General lower limb extension muscle strength was evaluated with the isokinetic mode of Strength Ergo 240 (SE240: Mitsubishi Electric Engineering Corporation, Tokyo, Japan) (19). The rotational speed was set at 50/min, and the backrest angle was set at 110 degrees during the measurement period. After orientation to strength measurement, the patients kicked pedals with five consecutive drives, and peak torque was calculated.

Handgrip force was measured using the CAMRY dynamometer (CAMRY EH101, Sensun Weighing Apparatus Group Ltd., Guangdong, China) in the sitting position with the humerus vertical and a 90-degree flexed elbow according to previous reports to avoid unexpected falls during the measurement (26, 27).

In addition to the assessments above, MDS-UPDRS part III item 7 toe-tapping was evaluated to clarify foot movement as a distal part of lower bradykinesia (22).

### Statistical analysis

All data were tested for normality using the Shapiro–Wilk test. To investigate the construct validity of the TIS, the relationships between TIS and MDS-UPDRS items, Mini-BESTest, BI, Strength of Extremities, and Hoehn and Yahr stage were analyzed using Spearman’s correlation coefficients. To analyze confounding factors within parameters other than the TIS, multiple regression analysis was performed. To analyze the relationships of background factors in each item on the TIS and other physical functions, factor analysis with Promax rotation was used. To determine the number of factors before the factor analysis, the Bayesian information criterion was applied to allow for more objective calculations even with a large sample size. Other determination methods such as the Kaiser-Guttman criterion and scree plot were not used in this study due to the risk of overestimation or uncertain features of visual judgment methods. Randomly occurring missing values were complemented using the multiple imputation method. All statistical analyses were performed with the statistical software R version 4.2.0 for Windows, and the significance level was set at *p* < 0.05.

## Results

**Figure 1:**
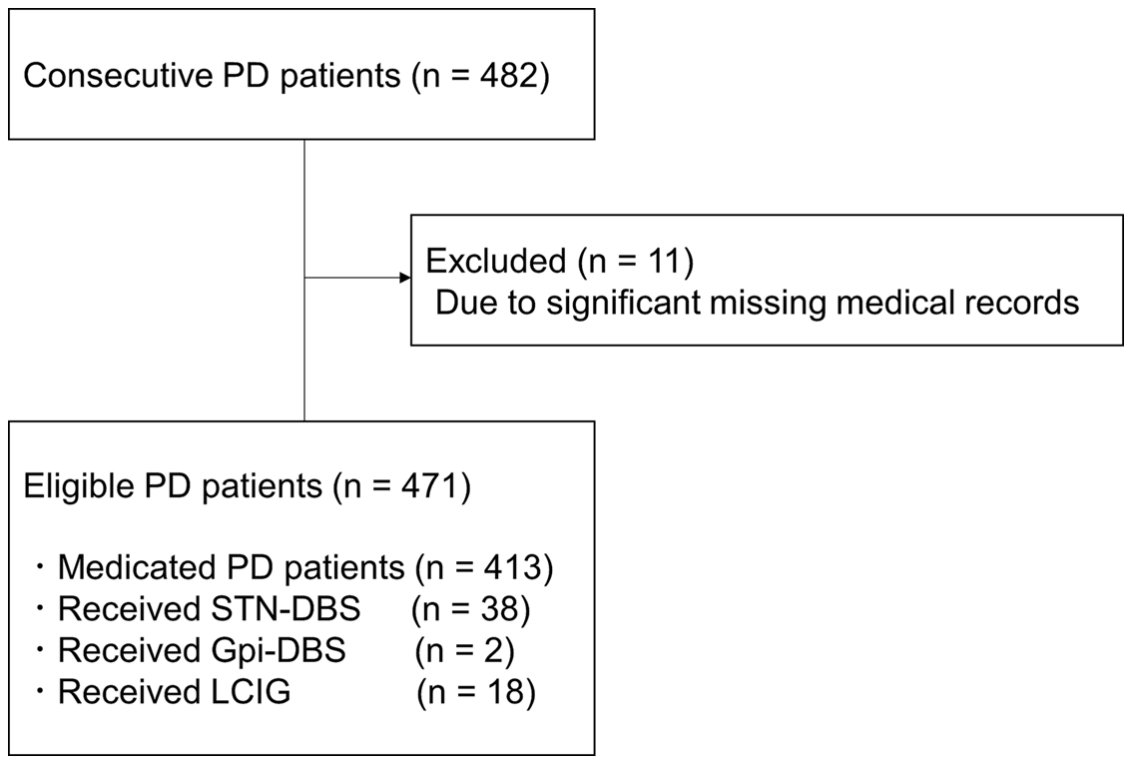
Schematic flow chart of study participants. PD, Parkinson’s disease; STN-DBS, Subthalamic Nucleus Deep Brain Stimulation; Gpi-DBS, Globus Pallidus Internus Deep Brain Stimulation; LCIG, Levodopa-carbidopa intestinal gel therapy.

After applying the exclusion criteria, data of 471 patients were selected for this study (Figure 1). Data of 11 patients with PD were excluded due to significant missing medical records.

Fifty-eight patients had previously received device-aided therapy, such as Subthalamic Nucleus Deep Brain Stimulation implantation (38 patients), Globus Pallidus Internus Deep Brain Stimulation implantation (2 patients), and Levodopa-carbidopa intestinal gel therapy (18 patients). Table 1 shows the demographic data of the eligible cases.

**Table 1:** Demographic data of the participants.

Age, years, mean (SD)	65.7 (10.6)
Sex, female/male, *n* (%)	237 (50.3)/234 (49.7)
Body height, cm, mean (SD)	159.9 (10.6)
Body weight, kg, mean (SD)	57.1 (40.8)
Body mass index, mean (SD)	22.2 (14.2)
Duration of disease, years, mean (SD)	12.1 (7.5)
Duration of medication, years, means (SD)	10.4 (8.2)
Hoehn and Yahr stage, median (IQR)	2.0 (1)
MDS-UPDRS part III, median (IQR)	22.0 (18.5)
Barthel Index, median (IQR)	80.0 (30.0)
Mini-BESTest total score, median (IQR)	19.0 (10.0)
Deep brain stimulation setting	
Pulse, microseconds, mean (SD)	54.9 (19.2)
Hz, mean (SD)	130.4 (40.1)
mA, mean (SD)	2.5 (0.9)
LDD, mg, mean (SD)	636.4 (947.8)
LEDD, mg, mean (SD)	283.5 (496.0)

Data are mean (SD), *n* (%), or median (IQR) values. SD, standard deviation; IQR, interquartile range; MDS-UPDRS, movement disorders society-unified Parkinson’s disease rating scale; Mini-BESTest, Mini-Balance Evaluation Systems Test; LDD, levodopa daily dose; LEDD, levodopa equivalent daily dose.

**Figure 2:**
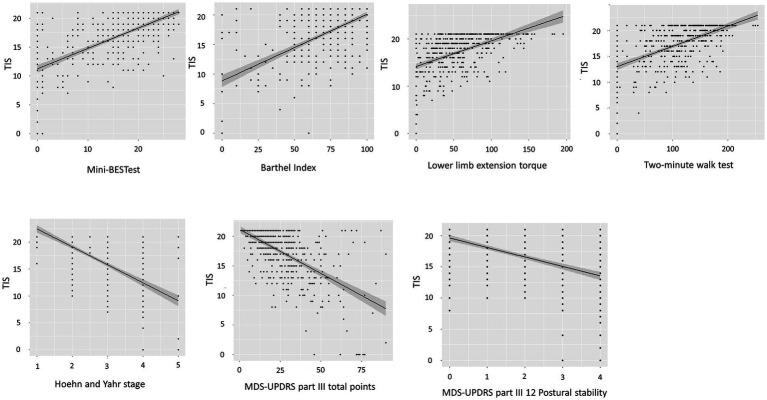
Scatter plots depicting the relationship between the TIS and associated assessment parameters. Spearman‘s correlation coefficients show a positive association of the TIS (range 0–21 points) and Mini-BESTest (range 0–28 points). Spearman’s correlation coefficients show negative associations of the TIS (range 0–21 points) and Hoehn and Yahr stage (range 1–5) and MDS-UPDRS part III total points (range 0–132 points). TIS, Trunk Impairment Scale; Mini-BESTest, Mini-Balance Evaluation Systems Test; MDS-UPDRS, Movement Disorders Society-unified Parkinson’s disease rating scale.

Spearman’s correlation coefficients showed moderate positive correlations between the TIS and the Mini-BESTest (*r* = 0.66, *p* < 0.001), Barthel Index (*r* = 0.57, *p* < 0.001), general lower limb extension torque (*r* = 0.51, *p* < 0.001), and the two-minute walk test (*r* = 0.54, *p* < 0.001). There were moderate negative correlations between the TIS and Hoehn and Yahr stage (*r* = −0.58, *p* < 0.001), MDS-UPDRS part III total points (*r* = −0.55, *p* < 0.001), and part III Postural stability (*r* = −0.51, *p* < 0.001) (Figure 2). In the other functions or general state, the TIS was weakly correlated with handgrip force (*r* = 0.46, *p* < 0.001), age (*r* = −0.37, *p* < 0.001), MDS-UPDRS part III toe-tapping (*r* = −0.34, *p* < 0.001), Arising from chair (*r* = −0.48, *p* < 0.001), Gait (*r* = −0.49, *p* < 0.001), Freezing of gait (*r* = −0.42, *p* < 0.001), and Posture (*r* = −0.45, *p* < 0.001). There were negligible correlations between the TIS and MDS-UPDRS part II Turning in bed (*r* = −0.29, *p* < 0.001), body mass index (*r* = 0.16, *p* = 0.007), duration of disease (*r* = −0.16, *p* < 0.001), duration of levodopa medication (*r* = −0.16, *p* < 0.001), and LEDD (*r* = 0.15, *p* = 0.0014).

Table 2 shows the results of multiple regression analysis between the TIS and age, body height, body weight, duration of disease, years, Barthel Index, LEDD, MDS-UPDRS II Turning in bed, MDS-UPDRS III total score, Hoehn and Yahr stage, Mini-BESTest total score, general lower extremity extension torque, two-minute walk test, and handgrip force. The analysis showed that the independent variables Barthel Index (*β* = 0.14, *p* = 0.01), MDS-UPDRS II Turning in bed (*β* = −0.12, *p* = 0.002), Hoehn and Yahr stage (*β* = −0.16, *p* = 0.003), and Mini-BESTest total score (*β* = 0.27, *p* = 0.00003) contributed significantly to the dependent variable TIS.

**Table 2:** Multiple regression analysis between the TIS total score and demographic and other clinical assessments.

	*β*	VIF	*p* value
Age	0.01	1.56	0.87
Body height	−0.01	1.58	0.73
Body weight	0.02	1.11	0.58
Duration of disease	0.02	1.08	0.63
Barthel index	0.14	2.75	0.01**
Levodopa equivalent daily dose	0.04	1.06	0.27
MDS-UPDRS II 9 Turning in bed	−0.12	1.36	0.002**
MDS-UPDRS III total score	−0.08	2.75	0.14
Hoehn and Yahr stage	−0.16	2.64	0.003**
Mini-BESTest total score	0.27	4.06	0.00003***
General lower extremity extension torque	0.01	3.18	0.93
Two-minute walk test	0.05	2.60	0.35
Handgrip force	0.07	2.39	0.16

*R*^2^ = 0.52, *p* < 0.0001. ^*^p < 0.05, ^**^p < 0.01. *β*, standardized partial regression coefficient; VIF, variance inflation factor; R2, multiple coefficient of determination; MDS-UPDRS, movement disorders society-unified Parkinson’s disease rating scale; Mini-BESTest, Mini Balance Evaluation Systems Test; TIS, Trunk Impairment Scale.

The Bayesian information criterion analysis yielded seven factors in the MDS-UPDRS part II Turning in bed, MDS-UPDRS III Toe tapping, PIGD score (Arising from chair, Gait, Freezing of gait, Postural stability, Posture), Mini-BESTest sub-score (Anticipatory postural adjustments, Automatic postural responses, Sensory orientation, and Dynamic gait), TIS sub-score (Perception of trunk verticality, Trunk rotation muscle strength on the right and left side, Righting reflex on the left and right side, Verticality, Abdominal muscle strength), general lower extremity extension torque, two-minute walk test, and handgrip force. The factor analysis with Promax rotation indicated that the first factor included the MDS-UPDRS II Turning in bed, MDS-UPDRS III Toe tapping, and PIGD score (Arising from chair, Gait, Freezing of gait, Postural stability, Posture). The second factor consisted of the Mini-BESTest sub-score (Anticipatory postural adjustments, Automatic postural responses, Sensory orientation, and Dynamic gait), and the two-minute walk test. The third factor comprised the TIS righting reflex on the left side, TIS righting reflex on the right side, and TIS abdominal muscle strength. The fourth factor included the TIS trunk rotation muscle strength on the right side and TIS trunk rotation muscle strength on the left side. The fifth factor involved the TIS perception of trunk verticality and TIS verticality. The sixth factor covered the general lower extremity extension torque, two-minute walk test, and handgrip force. The seventh factor included the MDS-UPDRS III Postural stability and Mini-BESTest Automatic postural responses (Table 3). All 7 factors showed moderate or low correlations with each other (Table 4).

**Table 3:** Factor loading matrix for 21 variables after promax rotation for the physical performance data.

	Factor 1	Factor 2	Factor 3	Factor 4	Factor 5	Factor 6	Factor 7
MDS-UPDRS II 9 Turning in bed	**0.51**	0.049	−0.071	−0.044	−0.122	−0.109	−0.197
MDS-UPDRS III 7 Toe tapping	**0.459**	−0.111	−0.034	0.022	−0.048	0.107	0.067
MDS-UPDRS III 9 Arising from chair	**0.882**	−0.114	−0.033	−0.005	0.065	−0.007	−0.084
MDS-UPDRS III 10 Gait	**0.843**	0.046	0.04	−0.021	0.033	−0.03	0.175
MDS-UPDRS III 11 Freezing of gait	**0.848**	−0.024	−0.048	−0.002	0.082	0.052	0.016
MDS-UPDRS III 12 Postural stability	**0.323**	0.124	−0.012	0.063	0.018	−0.098	**0.684**
MDS-UPDRS III 13 Posture	**0.533**	0.04	0.117	−0.009	−0.177	0.037	0.293
Mini-BESTest Anticipatory postural adjustment	−0.204	**0.776**	−0.015	−0.026	−0.021	−0.082	−0.062
Mini-BESTest Automatic postural responses	−0.035	**0.338**	0.024	−0.029	−0.016	0.054	**−0.435**
Mini-BESTest Sensory orientation	−0.101	**0.869**	−0.026	−0.023	0.043	0	0.112
Mini-BESTest Dynamic gait	−0.189	**0.746**	0.018	−0.002	−0.011	0.035	0.028
TIS perception of trunk verticality	0.042	0.04	0.023	−0.025	**0.781**	−0.009	0.061
TIS trunk rotation muscle strength on the right side	−0.014	−0.033	−0.134	**1.165**	−0.07	−0.023	0.067
TIS trunk rotation muscle strength on the left side	0.021	−0.003	0.139	**0.582**	0.104	0.02	−0.076
TIS righting reflex on the left side	−0.018	−0.014	**0.962**	−0.031	0.019	−0.025	−0.016
TIS righting reflex on the right side	−0.005	−0.02	**1.051**	−0.114	−0.023	0.005	0.009
TIS verticality	0.063	−0.012	−0.031	−0.052	**0.987**	−0.001	−0.014
TIS abdominal muscle strength	0.026	0.118	**0.334**	0.275	0.049	0.025	−0.078
General lower extremity extension torque	0.025	0.001	−0.032	−0.011	−0.014	**0.894**	−0.076
Two-minute walk test	−0.164	**0.462**	−0.042	0.034	−0.013	**0.315**	0.038
Handgrip force	−0.018	−0.065	0.028	−0.025	0.017	**0.821**	−0.011
Sum of squared loadings	3.19	2.31	2.21	1.80	1.67	1.62	0.87
Proportion of variance	0.15	0.11	0.11	0.09	0.08	0.08	0.04
Cumulative proportion of variance	0.15	0.26	0.37	0.45	0.53	0.61	0.65

Bold values indicate loadings > 0.30. MDS-UPDRS, movement disorders society-unified Parkinson’s disease rating scale; Mini-BESTest, Mini Balance Evaluation Systems Test; TIS, Trunk Impairment Scale.

**Table 4:** Factor correlations.

	Factor 1	Factor 2	Factor 3	Factor 4	Factor 5	Factor 6	Factor 7
Factor 1	1	0.444	0.646	−0.461	−0.56	−0.573	−0.55
Factor 2		1	0.585	−0.599	−0.64	−0.756	−0.641
Factor 3			1	−0.525	−0.687	−0.663	−0.546
Factor 4				1	0.52	0.694	0.601
Factor 5					1	0.602	0.493
Factor 6						1	0.702
Factor 7							1

## Discussion

The TIS was developed specifically to assess trunk function in patients with stroke. This is the first study to show that the TIS based on the motor function of the trunk and perceptual aspects had high construct validity. The present results showed that the TIS was correlated to disease severity, activities of daily living, limb strength, gait, and balance ability in patients with PD. Based on the results of correlation and multiple regression analysis, TIS may evaluate the trunk function related to balance function, disease severity, ADL function, lower limb strength, and which is different from the effects of age and BMI. The factors of the TIS were constructed with three different aspects (abdominal muscles and righting reflex component, perception and verticality component, and rotational component), and it differed from other factors that included clinical assessment items of Mini-BESTest, MDS-UPDRS part II Turning in bed, part III Postural instability and gait disturbance scores, two-minute walk test, and limb muscle strength. The TIS can be used to understand trunk impairment itself in patients with PD.

According to the previous studies, the factor analysis of TIS in patients with stroke did not divide the number of factors into multiple numbers, whereas multiple numbers were seen in patients with PD. The trunk impairment in stroke presents mainly with hypotonic hemiparesis, whereas PD presents with a variety of trunk impairments, including rigidity of trunk muscles (7), reduced righting reflexes (5), and altered vertical axis perception (5, 28). Although the results of this factor analysis might reflect the difference in the number of samples covered, there might be differences in background characteristics between stroke and PD.

After analyzing various factors such as MDS-UPDRS, Mini-BESTest, upper and lower extremity muscle strength, balance ability, and gait function, only the TIS was divided into three factors, even though these factors were correlated. Given that the Mini-BESTest is tailored explicitly to assess equilibrium in both standing and ambulatory scenarios, it was likely subjected to factor analysis as a single domain. The TIS administered in a supine or seated posture, and its broader assessment beyond mere motoric capacities of achievable or unattainable, might reflect why it was stratified into distinct domains during factor analysis. The elements that the TIS could capture separately were the [1] trunk righting reflex and abdominal muscle strength component, [2] the trunk rotation component, and [3] the verticality and vertical axis perception component, and it has been reported that, in PD, the trunk righting reflex and muscle strength (5), trunk rotation (9, 12), and vertical axis perception (5, 28) were decreased. The TIS in the present study is considered to be an assessment scale that is constructed to reflect these factors. The results of the factor analysis in the present study also suggest that the TIS may be an assessment index that can provide a more detailed evaluation of trunk dysfunction in patients with PD, which is difficult to obtain with other assessments.

In the clinical setting, trunk function is considered to be important for rolling over, but the TIS was not related to the MDS-UPDRS part II Turning in bed item in the present study. A previous study recruited only patients with PD with mild disease severity and showed that bed roll ability was related to trunk motor function, which was different from the result of the present study (6). One possible explanation is that these results might reflect the assessment method differences between the TIS, which reflects trunk dysfunction accurately by physical examination, and the MDS-UPDRS part II Turning in bed item, which allows turning movements even with compensatory movements, by questionnaire evaluation.

Previous studies have reported that trunk function in patients with PD affects ADL performance (6, 12), balance function, and gait function (7, 16). In the present study, the direct impact on trunk function on multiple regression analysis resulted in a low contribution rate, as indicated by the standard partial regression coefficients, although there were significant differences. Therefore, it is unlikely that ADL, balance function, and walking ability have a strong influence on trunk function due to these factors. From a clinical perspective, although ADL, balance function, and walking ability are correlated with trunk function, it is unlikely that they are confounding factors that influence trunk function. Rather, it is possible that trunk function influences ADLs, balance function, and walking ability.

Evaluation scales that separately quantify trunk function, balance function, and gait function in patients with PD have been scarce, making it difficult to capture the relationships among trunk function, balance function, and gait function in patients with PD, as well as trunk function itself. Since the TIS can comprehensively capture the trunk function of patients with PD from the motor and perceptual aspects, the clinical application of this assessment is considered highly meaningful.

The present study has some limitations. First, because this was a cross-sectional study, predictive factors of TIS itself or effect for the other physical functions such as balance function, and minimum clinically important difference could not be analyzed. Therefore, an additional cohort study should be planned. Second, the subjects in this study were only clinically diagnosed with PD, not pathologically confirmed. Third, this study did not contain the aspect of nonmotor symptoms and fluctuations of dopaminergic medications. A future study should include the assessment of nonmotor features and fluctuations of dopaminergic medications. In addition, it is necessary to conduct randomized, controlled studies to clarify the causal relationship to determine whether the improvement in trunk function captured by the TIS analyzed in the present study leads to an improvement in physical function in patients with PD.

## Conclusion

The TIS can evaluate three different components including motor and perceptual aspects of trunk function separately from limb muscle strength, balance function, and parkinsonism. These findings suggest that the TIS can be useful for exploring the underlying trunk impairment as a basis for activities of daily living, gait function, and balance ability in patients with PD.

## Data availability statement

The raw data supporting the conclusions of this article will be made available by the authors, without undue reservation.

## Ethics statement

The studies involving humans were approved by Research Ethics Committee, Faculty of Medicine, Juntendo University. The studies were conducted in accordance with the local legislation and institutional requirements. The ethics committee/institutional review board waived the requirement of written informed consent for participation from the participants or the participants’ legal guardians/next of kin because informed consent was obtained in the form of an opt-out on the website.

## Author contributions

KS: Conceptualization, Data curation, Formal analysis, Investigation, Methodology, Software, Validation, Writing – original draft, Writing – review & editing. YY: Data curation, Investigation, Writing – review & editing. YK: Data curation, Investigation, Writing – review & editing. KW: Data curation, Investigation, Writing – review & editing. EK: Data curation, Investigation, Writing – review & editing. KHar: Writing – review & editing. YTa: Writing – review & editing. YF: Writing – review & editing. TY: Writing – review & editing. TM: Writing – review & editing. HM: Writing – review & editing. RI: Writing – review & editing. YM: Writing – review & editing. MT: Writing – review & editing. KHo: Writing – review & editing. KHat: Writing – review & editing. YO: Writing – review & editing. YTo: Writing – review & editing. TH: Writing – review & editing. NH: Project administration, Supervision, Writing – review & editing. TF: Methodology, Project administration, Supervision, Writing – review & editing.

## Funding

The author(s) declare that no financial support was received for the research, authorship, and/or publication of this article.
